# Rehabilitation in Animal Models of Stroke

**DOI:** 10.1298/ptr.R0022

**Published:** 2023-04-29

**Authors:** Mushfiquddin KHAN

**Affiliations:** ^1^Professor Emeritus, Department of Pediatrics, Charles P. Darby Children’s Research Institute, Medical University of South Carolina, USA

**Keywords:** Stroke, Rehabilitation, Neuroprotection, Neurorepair, Functional recovery

## Abstract

Objective: The purpose of this review was to evaluate the efficacy of rehabilitation strategies in animal models of stroke and their correlation with human stroke studies. Methods: General description of a stroke, functional recovery, and rehabilitation modalities were included from published studies in the field of animal models of cerebral ischemia and ischemia–reperfusion. Results: In stroke survivors, rehabilitation plays a significant role to improve motor function, cognition, and other subtle behaviors. Targeted pharmacological agents, including neuroprotective drugs, are helpful in animal models of stroke. However, no drug has yet been found that meets the criteria that would make it the Food and Drug Administration-approved treatment for human stroke. Instead, the rehabilitation of stroke in humans is limited to physical and occupational therapy, speech therapy, environmental enrichment, and social activities, as well as spiritual and family support. Conclusion: Studies on stroke injury and the significance of stroke animals’ rehabilitation, including physical and pharmacological, approaches are highlighted.

**S**troke is the major cause of long-term disability worldwide. Stroke survivors suffer from long-term disability due to motor, cognitive, and subtle behavior deficits^1^^,^^2)^. Varying degrees of functional deficits are prevalent for long even in patients after successful recanalization by stent-retriever devices^3)^ or thrombolysis by recombinant tissue plasminogen activator^4^^,^^5)^, indicating that the restoration of cerebral blood flow is not enough for functional recovery^6^^,^^7)^. Reperfusion-induced functional deficits provide a renewed opportunity to test the efficacy of rehabilitation in animal models of transient cerebral ischemia followed and reperfusion (IR) because it mimics the stroke injury in humans after thrombolysis or endovascular thrombectomy (recanalization). Therefore, the discussion in this review will focus mainly on the animal model of transient cerebral ischemia/hypoxia and reperfusion.

Stroke comes from damaged blood vessels in the brain. The blood vessels become blocked (called “ischemic” stroke) due to a blood clot, fat deposits, or simply because the vessels become thick and hard. Sometimes, these blood vessels burst (called “hemorrhagic” stroke). An ischemic stroke cuts off the brain’s oxygen and nutrients either for a short (transient stroke) or a long time (permanent stroke). The most common type of stroke, ischemic, affects nearly 700,000 people per year and the prevalence of stroke increases with the increasing aged population in the US^8)^. Millions of people have to adapt to an altered life with incurable restrictions in their daily activities. Many must depend on others simply to survive. The direct annual economic burden of the stroke-related cost was projected from $71.6 billion in 2013 to $184.1 billion in 2030^9)^.

The ability to forecast stroke is critical; however, stroke by nature is unpredictable. The vascular changes that precede stroke develop stealthily and are not evident for a long time. Some of stroke risk factors include hypertension, metabolic syndrome, physical inactivity, obesity, high-fat diet, being single, being unhappy, anxiety, smoking, and being born in the wrong demographics. However, their link and the stroke-inducing deleterious mechanisms are not understood. Although stroke is not considered a genetic (inherited) disorder; however, stroke association with epigenetic (metabolome) is regarded valid. A disturbed metabolome is also observed in several peripheral organs including spleen, kidney, gut, and liver, and their function is linked with the severity of stroke. The irony is that neither an effective brain repair therapy nor metabolic disturbances after stroke are available mainly due to a limited understanding of multifactorial injurious stroke mechanisms. Furthermore, recovery from stroke injury is also restricted due to symptoms of anxiety, depression, fatigue, and pain^10^^–^^13)^.

In developed countries, the incidence of stroke is declining, largely due to efforts to lower blood pressure and reduced smoking. However, the overall rate of stroke remains high due to the increase in the aging population. Nearly three-quarters of all strokes occur in people over the age of 65. The risk of having a stroke more than doubles each decade after the age of 55 years. Stroke is equally or more prevalent in aged women compared to aged men^14)^. Younger females (before menopause) are significantly protected from stroke due likely to female sex hormones including estrogen. Most of the therapeutic approaches and rehabilitation modalities following stroke are common to both genders. Stroke in children is also among the top ten causes of death and carries often lifelong morbidity^15)^. While neurons lose neuronal function in adults, neurons in children are unable to gain the function following stroke. The societal burden of childhood stroke is likely to be greater than in adults because children surviving stroke face many more years living with a disability. Both diagnosis and treatment of infant and childhood stroke are difficult^16)^. Childhood stroke is caused by factors such as infection, trauma, cardiac, hematologic disorders, arteriovenous malformation, and brain tumor. A sudden compromise of neurological symptoms such as speech disturbance, limb incoordination, ataxia, headache, and altered consciousness are indicative of stroke in children and immediate medical care including thrombolytic/neuroprotective therapy is required^17)^.

In adult stroke survivors, rehabilitation using pharmacological drugs and/or physical activities plays a significant role in improving movement, cognition, and subtle behaviors as depicted in Figure 1. Targeted pharmacological agents including neuroprotective drugs such as minocycline^18)^, S-nitrosoglutathione^19^^–^^21)^, sodium nitrite^22)^, and many more^23)^ are successful in animal models of stroke. However, their efficacy and clinical relevance do not meet the criteria to be approved for stroke treatment. In addition to pharmacological drugs, appropriate cell infusion, therapeutic exercises, environmental enrichment, social activities, family support, and spiritual leanings also aid and accelerate functional recovery^8^^,^^24^^–^^26)^.

**Fig. 1.**
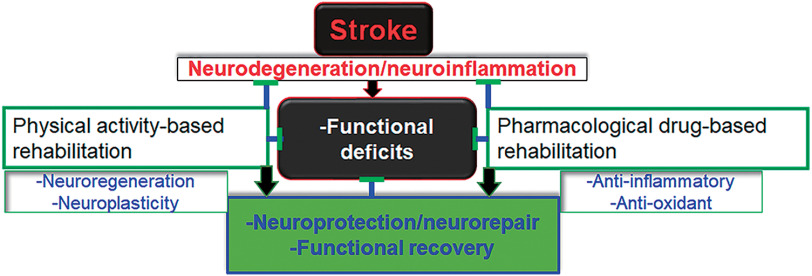
Schematic showing key mechanisms of the effects of rehabilitation on animal models of stroke

Stroke (permanent ischemia or transient ischemia and reperfusion) results in oxidative burst and the activation of devastating neuroinflammatory cascades leading to neurodegeneration. If untreated, it also obstructs the mechanisms of neural regeneration and brain repair in the chronic phase of the injury. Combined effects of injury mechanisms in the acute and chronic phases lead to profound functional deficits. Functional outcomes can be improved by different rehabilitation strategies including physical-activity-based modalities and pharmacological drug-based treatment regimens.

Animal research demonstrates that treatment with many neuroprotective or neurorepair agents has not provided clinically beneficial effects mainly due to the limitation of animal models (young versus aged, and permanent ischemia versus ischemia–reperfusion), biological variables, and limited understanding of differential stroke’s secondary injury mechanisms in the acute versus chronic phase of stroke injury. For functional recovery, clinical trials show that neuroprotective drugs failed due to the lack of efficacy in the chronic phase^27^^,^^28)^. Therefore, an ideal therapy must ameliorate acute as well as chronic phases by well-understood mechanisms.

The rehabilitation approach is essential for stroke survivors to stimulate the recovery of functions. Therefore, improvement by a therapeutic agent or rehabilitation in neurorestoration and neurobehavioral functions in animal models determines the efficacy and the clinical relevance of therapy in human stroke (Figure 1). Physical exercise-induced functional recovery in animal models of stroke translates into recovery in many stroke patients. Running exercise is widely used for the rehabilitation of stroke animals.

## Justification of running on wheels motor training for physical exercise

Running on wheels resembles natural conditions more closely than any other physical modality. It promotes neurorestorative activity, decreases the mortality rate, and shows an efficient recovery of neuromotor functions following stroke^29)^. Moreover, motor exercise is also reported to upregulate brain-derived neurotrophic factor expression^21)^ as well as to reduce the decline in cognitive function^30)^. Therefore, running on wheel modality for exercise using a walking wheel bed model is frequently used in animal stroke studies. In addition, rotarod, treadmill, and environmental enrichment approaches for physical exercise-based activities are common in animal models of stroke. In such studies, the severity of stroke and the degree of exercise are confounding factors. To determine the quantifiable recovery with time by rehabilitation strategies, several tasks/tests relevant to human stroke are performed in animal models of stroke as described in the following.

## Evaluation of functional recovery in rat/mouse models of stroke

1) Modified neurological severity score (mNSS)

Neurological function measurements are performed as previously described from our laboratory^31)^. The test is sensitive to unilateral cortical injury because it reflects multiple asymmetries, including postural, sensory, and forelimb and hindlimb use asymmetries. A detailed description of this functional test has been previously reported^32)^. In our studies, mNSS is scaled from 0 to 12 with 0 as normal and maximal deficit score as 12^19)^, which reflects combined sensorimotor, beam balance, and reflex abnormal movement functions^31)^.

2) Foot-fault test

Animals are tested for forelimb movement dysfunction while walking on elevated metal grids with randomly missing support bars. With each weight-bearing step, the forelimb can fall or slip between the metal support bars, which is recorded as a foot fault. The total number of forelimb steps and the total number of foot faults are recorded as described^33)^.

3) Cylinder test

Exploration is a natural behavior of rodents. The cylinder test is used to determine rodents’ exploratory behavior using sensorimotor function following stroke^34)^. It measures forelimb asymmetry in experimental animals. The asymmetry causes behavioral deficits in the contralateral forelimb of the injured IR animals. Animals reared onto their hind limbs and touched the cylinder wall with forelimb placement to balance themselves while exploring their surroundings. The animal is placed in a Plexiglas cylinder and videotaped as described. Touches of both affected and unaffected paws and paw-dragging are analyzed^20)^.

4) Forelimb placing test

The forelimb placing test scores the animals’ ability to place their forelimb on a tabletop in response to whisker, visual, tactile, or proprioceptive stimulation. The test reflects function and recovery in the sensory-motor systems. Animals are held by their torsos with forelimbs hanging freely. A score of 1 is given each time the rat places its forelimb on the edge of the tabletop in response to the vibrissae stimulation. Percentage of successful placing responses are determined (number correct × 10). The lateral tactile placing is similar to the whisker placing, except that the placing response is induced by gently contacting the lateral side of the forelimb to the edge of the tabletop, whereas forward tactile placing is induced by contacting the frontal side of the forelimb to the edge of a tabletop. The scale is scored as described^35)^.

5) Body swing test

This test reflects the symmetry of striatal function^19^^,^^36)^. A normal animal typically has an equal number of swings to the contralateral side. Each rat is held along the vertical axis (defined as no more than 10° to either the left or the right side) approximately one inch from the base of its tail and elevated an inch above a table surface. A swing is recorded whenever the rat moves its head out of the vertical axis to either side. The animals have to return to the vertical position for the next swing to be counted.

6) Adhesive removal test

The adhesive removal task is a test to assess somatosensory function and is thus used to highlight minute deficits. The test is also useful to assess even smaller recovery since it is capable of measuring long-lasting deficits. Finally, it allows for longitudinal studies through adaptation of the size of the adhesive tape according to the age of the individual tested^37)^. Two adhesive tapes are applied with equal pressure on each animal paw. The order of placement of adhesive (right or left) is alternated between each animal. The time to contact and to remove each adhesive test is recorded, with a maximum of 2 min as described^31)^.

*7)* Motor function tests by rotarod task

Fine motor coordination is evaluated using a rotarod task. This task is a reliable test to evaluate short-term vestibulomotor function and is widely used in animal models of IR and traumatic brain injury^38^^–^^40)^. Animals are trained on an automated 4-lane rotarod unit. Each animal is given 3 trials, and the mean latency of the three are calculated. Motor function tests by rotarod studies are complemented by the beam walk task as previously described^21^^,^^41)^.

8) Learning/Memory tests (Morris water maze [MWM])

Spatial learning and memory deficits in rodents are investigated using a water maze paradigm^38^^,^^42^^,^^43)^ similar to that originally described by Morris^44)^ and extensively used in experimental stroke. Spatial learning is assessed by training the animals to locate a hidden, submerged platform using extra maze visual information. It should be noted that a variety of parameters and outcomes are measured in this computerized video system, including swim speed, latency to find the platform, path length, and percentage of time in each quadrant. Differences in swimming speed indicate differences in motor function, whereas path length (time and distance to find platform) measurements determine cognitive processes as described^45)^. Working memory is also examined by comparing repeated trials on the same day^38)^. To minimize the confounding effects of motor deficits on spatial learning/memory and to complement with MWM data, a well-recognized memory test “Novel Object Recognition” (NOR) is used.

9) Memory test (NOR)

This test determines nonspatial hippocampal-mediated memory and is based on an animal’s spontaneous ability to explore a novel object. The advantages of the test is that it is independent of motor function and it is quick and simple to perform^46)^. Animals are habituated twice for 5 min to the NOR apparatus to familiarize the testing environment. On the test day, each animal is again allowed to explore two identical objects for 5 min (trial phase). After resting for 4 h, the animal is brought to the box and allowed to explore a familiar and a novel object for 5 min. Both trials and test phases are recorded and analyzed. Object exploration is defined as the animal sniffing or touching the objects but not by leaning against, standing on, turning around, or sitting on the objects. Discrimination index ([time spent exploring the novel object-time spent on exploring the familiar object]/total exploration time) is calculated for each of the animals during the test phase as previously described^20^^,^^47)^.

10) Gait analysis

CatWalk system gait analysis has been validated in neuroscience research and experimental procedures for several neurological disorders including stroke, and it is an excellent method for tracking gait disruption with time due to stroke injury^48)^. The Catwalk system represents a rapid and sensitive way to objectively quantify several gait parameters such as position, pressure, and surface area of each paw. The mouse traverses a glass plate voluntarily (toward a goal box), while its footprints are captured by video. The software with the system subsequently visualizes the prints and calculates statistics related to print dimensions and the time and distance relationships between footfalls.

## Conclusions

Stroke is associated not only with significant mortality but also with morbidity, dementia, depression, fatigue, pain, as well as a great financial burden. Unfortunately, an effective therapy neither for neuroprotection nor for functional recovery (rehabilitation) following stroke is available. Although a large number of rehabilitation modalities are available for human stroke, many of them are much less effective due to social and personal factors and varying severity of injury in stroke patients. To advance the field and to investigate more relevant therapy to stroke in humans, rehabilitation studies using neuroprotective drugs and physical activities in animal models of stroke are required to continue using new and novel cellular/molecular mechanism-based approaches. A combination of both strategies is anticipated to provide greater and accelerated rehabilitation in stroke survivors.

## Acknowledgments

This work was supported by grants from the U.S. Department of Veterans Affairs (RX002090) and the National Institute of Health (R21NS114433). We thank Drs. Inderjit Singh, JeSeong Won, and Fei Qiao for their input in the preparation of the manuscript.

## Conflict of Interest

The author declares that he has no conflict of interest.
